# Degradation of neutrophil extracellular traps co-varies with disease activity in patients with systemic lupus erythematosus

**DOI:** 10.1186/ar4264

**Published:** 2013-08-14

**Authors:** Jonatan Leffler, Birgitta Gullstrand, Andreas Jönsen, Jan-Åke Nilsson, Myriam Martin, Anna M Blom, Anders A Bengtsson

**Affiliations:** 1 Lund University, Department of Laboratory Medicine Malmö, Section of Medical Protein Chemistry, Inga Marie Nilssons gata 53 floor 4, 205 02 Malmö, Sweden; 2 Lund University, Department of Laboratory Medicine Lund, Section of MIG, Sölvegatan 23, 223 62 Lund, Sweden; 3 Lund University, Department of Clinical Sciences, Section of Rheumatology, Kioskgatan 3, 221 85 Lund, Sweden

**Keywords:** Systemic lupus erythematosus, neutrophil extracellular traps, degradation, glomerulonephritis, prospective study

## Abstract

**Introduction:**

The ability to degrade neutrophil extracellular traps (NETs) is reduced in a subset of patients with systemic lupus erythematosus (SLE). NETs consist of chromatin covered with antimicrobial enzymes and are normally degraded by DNase-I, an enzyme which is known to have reduced activity in SLE. Decreased ability to degrade NETs is associated with disease activity. In the current study we investigated how the ability of serum from SLE patients to degrade NETs varies during the course of SLE as well as what impact this may have for the clinical phenotype of SLE.

**Methods:**

Serum from 69 patients with SLE, included in a prospective study, was taken every 60 days for a median of 784 days. The ability of serum to degrade NETs was determined and associated with clinical parameters occurring before and at the time of sampling, as well as after sampling by using conditional logistic regression.

**Results:**

As many as 41% of all patients in the study showed decreased ability to degrade NETs at least once, but with a median of 20% of all time points. Decreased degradation was associated with manifestations of glomerulonephritis as well as low complement levels and elevated levels of antibodies directed against histones and DNA. Furthermore, the odds ratio for the patient to develop alopecia and fever after an episode of decreased NETs degradation was increased by four to five times compared to normal.

**Conclusions:**

Decreased degradation of NETs is associated with clinical manifestations in SLE and may contribute to disease pathogenesis. Potential therapeutics restoring the ability to degrade NETs could be beneficial for certain patients with SLE.

## Introduction

The autoimmune disease systemic lupus erythematosus (SLE) is a complex and heterogeneous disease with the patients displaying a variety of symptoms ranging from glomerulonephritis to skin rashes and chronic fatigue. A common feature of SLE is the generation of anti-nuclear antibodies. It has been hypothesized that SLE evolves from the inefficient or improper clearance and degradation of dying cells [1-4]. Numerous genes have been associated with the disease, spanning from immune modulatory genes to complement factors [5], all crucial to ensure a proper immune response and efficient clearance of apoptotic and necrotic cells. In 2004, a new potential antigen source in SLE was discovered with the description of neutrophil extracellular traps (NETs) [6]. NETs consist of chromatin and antimicrobial enzymes that are released from neutrophils as a "last-resort" defense to trap and kill pathogens. It was subsequently shown in two independent studies that NETs are efficiently degraded in serum from healthy controls, whereas this ability is reduced in a subpopulation of SLE patients [7,8]. The patients with decreased ability to degrade NETs suffered from a severe form of SLE with glomerulonephritis and additionally exhibited autoantibodies that recognized NETs. Numerous recent reports further show involvement of NETs in SLE. This spans from how NETs are more easily formed by neutrophils isolated from SLE patients, potentially through elevated interferon-α levels or the presence of activating antibodies in these patients to how non-degradable complexes of chromatin and antimicrobial peptides are found in SLE sera [9]. Together, this all could contribute to the tissue damage in SLE [10]. It has long been known that SLE patients display a decreased ability to degrade DNA [11] and there are many theories why this is the case. DNase-I is the enzyme responsible for degradation of NETs and it is inhibited by globular actin. Actin may be released by platelets, and dying cells during inflammation [12] and has also been shown to prevent excessive chromatin degradation in apoptotic and necrotic cells [13]. Further, autoantibodies against DNA could shield the DNA from DNase-I and have additionally been described to cross react directly with the enzyme potentially leading to inhibition [14]. We also showed that C1q binds to NETs and prevents degradation [8], indicating formation of non-degradable complexes on NETs consisting of autoantibodies and complement. Interestingly, in our previous study we observed that the decreased ability of serum from SLE patients to degrade NETs is mostly not permanent but changes between time points with different disease activity [8]. To thoroughly determine the extent of this phenomenon, we used serum samples from a prospective study where 69 patients with SLE were followed for up to five years with samples taken approximately every two months. At each sampling, we measured the ability of patient serum to degrade NETs. Clinical manifestations, laboratory variables and treatments were registered at all time-points in the patients and these variables were used to determine temporal associations with decreased ability to degrade NETs. We identified a number of distinct clinical manifestations and laboratory variables that preceded the time-point of decreased NET degradation and some that appeared at the same time-point as well as after the time-point of decreased NET degradation.

## Materials and methods

### Patients and sample preparation

Sixty nine patients (6 males and 63 females) with a majority of Caucasian origin, except for two with Asian and one of Hispanic origin, with a median age of 39 (range 18 to 76) with established SLE, fulfilling at least four or more American College of Rheumatology (ACR) 1982 classification criteria for SLE [15] were recruited to this prospective study at the Department of Rheumatology, Skåne University Hospital in Lund (Sweden). The distribution of ACR classification criteria for SLE of the patients is described in Table 1. The overall aims of the prospective follow-up program were partly to improve care by close monitoring of SLE-patients at risk of complications and partly to identify risk factors, clinical and laboratory variables, for disease exacerbations. Disease activity was recorded at every visit using the SLE Disease Activity Index 2000 (SLEDAI-2K) [16] together with infections and other clinically relevant information. Visits were scheduled every two months with a range of 46 to 74 days, depending on disease activity. The median study duration for the patients was 784 days (mean 921 days) with a range of 139 to 1,792 days corresponding to a median of 14 (2 to 43) visits. Only visits within 46 to 74 days were used for temporal associations. Sera from 77 healthy volunteers (15 males and 62 females) with a median age of 42 (range 19 to 77) were used as controls. All patients and healthy controls gave informed consent to participate in the study, which was approved by the local ethics committee (Lund University) according to the Helsinki declaration.

**Table 1:** ACR classification criteria of patients included in study

ACR classification criteria	Patient number (%)
**Malar rash**	46 (67%)
**Discoid rash**	18 (26%)
**Photosensitivity**	42 (61%)
**Oral ulcer**	16 (23%)
**Arthritis**	55 (80%)
**Serositis**	35 (51%)
**Nephritis**	35 (51%)
**Neurological disorder**	3 (4%)
**Hematological disorder**	38 (55%)
**Immunological disorder**	53 (77%)
**Anti-nuclear antibodies**	69 (100%)

Patient characteristics according to ACR classification criteria. ACR, American College of Rheumatology

### Isolation of neutrophils

Neutrophils were isolated from healthy volunteers according to a previously published method [17]. Briefly, blood from a healthy volunteer was separated by centrifugation on a Histopaque 1119 column (Sigma-Aldrich, St Louise, MO, USA), the granulocyte-rich fraction was isolated, washed and neutrophils were isolated by centrifugation on a Percoll gradient (85 to 65%) (GE Healthcare Biosciences, Uppsala, Sweden) and isolated from the intersection of the 75% and 70% layers, washed and resuspended in RPMI with 10 mM Hepes at 1 × 10^6 ^cells/ml. Purity of neutrophils (>80%) was determined by forward-side scatter analysis in combination with surface marker expression defined as CD14^low^/CD15^+^/CD16^+ ^by PE-labeled anti-CD14 (BD), FITC-labeled anti-CD15 and APC-labeled anti-CD16 (both from Immunotools, Friesoythe, Germany) in a CyFlow Space (Partec, Münster, Germany)

### Generation and degradation of NETs

Freshly isolated neutrophils from healthy volunteers, 50,000/sample, were seeded onto a 96-well flat bottom plate (Nunc, Thermo Fisher Scientific Inc, Waltham, MA, USA) with 20 nM PMA (Sigma-Aldrich) for 4 h at 37°C and 5% CO_2 _to generate NETs. After incubation, cell medium was removed and 10% patient sera, control sera or sera with 5 to 320 μg/ml hydroxychloroquine (Sigma-Aldrich) in 10 mM Tris-HCl, pH 7.5, 50 mM NaCl, 10 mM MgCl_2 _and 2 mM CaCl_2 _were added to NETs and incubated for 60 minutes at 37°C. During this time, degraded NETs were released into solution. Aliquot of the solution was then transferred to PBS with a final concentration of 2 mM EDTA to stop further degradation and DNA content was quantified using picoGreen (Invitrogen, Grand Island, NY,) according to the manufacturer's instructions. As an internal control, pooled normal human serum was used and all samples were compared to the mean of the internal controls for each individual experiment. All samples were measured twice, first in duplicates followed by once in singlets and the mean of the two measurements was used for analysis.

### Clinical laboratory assays

Routine laboratory testing at Department of Clinical Immunology (Lund University Hospital, Lund, Sweden) was used to determine levels of hemoglobin, white blood cell count, neutrophil granulocytes, platelets, and levels of anti-DNA (*Crithidia luciliae *immunofluorescence test) and anti-cardiolipin titers as well as C-reactive protein (CRP), creatinine, C1q, C3 and C4. Titers of anti-histone antibodies were determined as previously described [18].

### Reference values and medications

For the following variables, reference values used in clinical routine were applied to construct categorized variables, anti-cardiolipin >20 U/mL, low hemoglobin <120 g/L, low serum creatinine is based on individual reference values according to Gault and colleagues [19], low C1q <77% of normal serum pool, low C3 <0.77 g/L, low C4 <0.12 g/L. For cell counts the following values were used: leukopenia <3 × 10^6 ^cells/ml, neutropenia <1.5 × 10^6 ^cells/ml and lymphopenia <1.5 × 10^6 ^cells/ml. For medications, high corticosteroid dosage was defined as dosages above 20 mg/day of prednisone and use of immunosuppressants include: cyclophosphamide, mycophenolate mofetil, azathioprine, cyclosporine A and methotrexate.

### Statistical analysis

To determine statistical significance of difference for continuous data among three groups or more, the Kruskal-Wallis test was used followed by a post-test using Dunn's method for comparisons with control. To determine significant differences for nominal data between two groups the χ^2 ^test was used. All analyses were carried out using JMP 7 and 9 (SAS Institute, Cary, NC, USA). Association of clinical parameters with a decreased ability to degrade NETs was determined using conditional logistic regression with patient as strata variable to handle repeated measurements within subject, on SAS system 9.3 (SAS).

## Results

**Figure 1:**
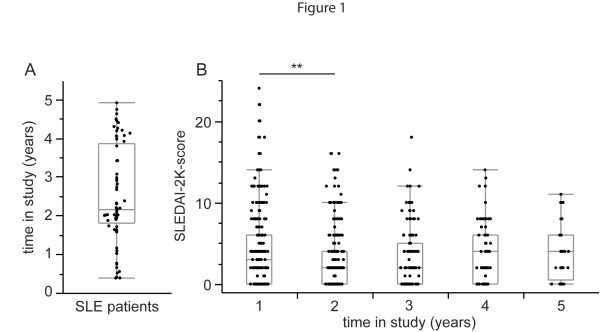
**Patient SLEDAI-2K decreased during the study**. **A**. Duration of time (in years) during which the patients were included in the study. Quantiles are shown in gray with a median of 2.1 years (784 days) and mean of 921 days (not shown). **B**. Individual SLEDAI-2K scores recorded from patients during the study, divided into intervals of one year from study inclusion. Significance of difference of values compared to the SLEDAI-2Ks during the first year was calculated using Kruskal-Wallis test followed by Dunn's post test and is displayed as **, *P *< 0.01. SLEDAI-2K, Systemic Lupus Erythematosus Disease Activity Index 2000

Serum samples of SLE patients were taken every two months for a median of 784 days (mean 921 days). The duration of time for which each patient was included in the study is displayed in Figure 1A. The disease activity as measured by SLEDAI-2K scores, recorded from the patients at each time point, improved significantly after two years from the study inclusion (Figure 1B).

**Figure 2:**
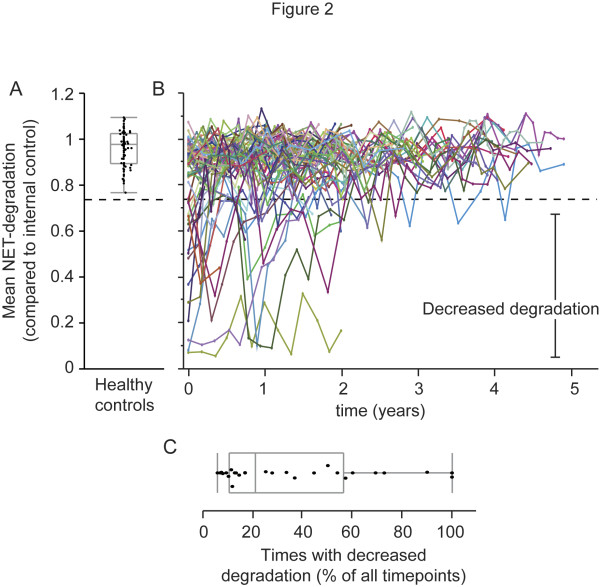
**Decreased ability to degrade NETs affects 41% of the SLE patients**. **A-B**. The ability of serum to degrade NETs compared to internal control of serum from 77 healthy controls (A) and 69 patients with SLE followed during multiple time points (B). Dashed line indicates cut-off for "normal" and "decreased" degradation. **C**. The fraction of all time points for which patients, who at least once displayed a decreased ability to degrade NETs, had a decreased NET degrading ability. Quantiles are displayed in gray with a median of 20% and mean of 34% (not shown). NETs, neutrophil extracellular traps; SLE, systemic lupus erythematosus

The presence of auto antigens, such as NETs, in SLE fuels the inflammation contributing to the disease. Therefore, we measured the ability of the patient's sera to degrade NETs at each time point of serum collection throughout the study. To determine the normal range of the ability to degrade NETs, sera from 77 healthy volunteers were used (Figure 2A). A cut-off was set as previously described [8] to three standard deviations below the mean NET-degradation of the controls and thus defined "normal" and "decreased" ability to degrade NETs. The NET-degrading abilities were subsequently measured in all patient samples and displayed over time, starting at study inclusion (Figure 2B). From all measurements recorded, a significant (*P *= 0.02) improvement was observed in the third year after study inclusion (data not shown), but varied highly for individual patients. To quantify how common an episode with decreased ability to degrade NETs is, the number of SLE patients whose sera never showed decreased NET degradation was compared with sera that showed decreased degradation at least once. We observed that 28 (41%) patients, at least once, displayed a decreased ability to degrade NETs whereas the remaining 41 patients (59%) never displayed decreased degradation. Out of the 28 patients whose sera at least once exhibited a decreased ability to degrade NETs, two had a reduced ability 100% of the times (13 and 4 time points, respectively), whereas the median frequency was at 20% (mean 34%) of all time points (Figure 2C). Together this shows that the ability to degrade NETs may vary for each patient over time and also that a decreased ability to degrade NETs is a rather common feature of SLE and corresponds well to previous findings [7,8].

The ACR classification criteria for SLE describe cumulatively the history of a patient's clinical manifestations since the onset of disease and give valuable information of the clinical phenotype. To determine if there was a certain clinical subgroup of patients who at least once displayed a decreased ability to degrade NETs compared to patients who never did, the ACR classification criteria of the two groups were compared (Table 2). Patients who at least once exhibited a reduced NET degradation during the study more often had a clinical history of antibodies against DNA. These patients with decreased NET degrading ability also showed an inverse association with a history of photosensitivity, a symptom associated with a less severe form of SLE.

**Table 2:** ACR classification criteria for patients with normal and decreased ability to degrade NETs

ACR classification criteria	Normal (*n *= 41) number (%)	Decreased at least once (*n *= 28) number (%)	*P*-value (Pearson)
**Malar rash**	27 (66%)	19 (68%)	0.86
**Discoid rash**	12 (30%)	6 (21%)	0.47
**Photosensitivity**	**30 (73%)**	12 (43%)	0.01*
**Oral ulcer**	8 (20%)	8 (29%)	0.38
**Arthritis**	32 (78%)	23 (82%)	0.68
**Serositis**	18 (44%)	17 (61%)	0.17
**Nephritis**	19 (46%)	16 (57%)	0.38
**Neurological disorder**	2 (5%)	1 (4%)	0.79
**Hematological disorder**	21 (51%)	17 (61%)	0.44
**Immunological disorder**	27 (66%)	**26 (93%)**	0.009*
***Anti-dsDNA***	22 (54%)	**23 (82%)**	0.01*
***Anti-Sm***	4 (10%)	3 (11%)	0.90
***False positive Wasserman***	5 (12%)	1 (4%)	0.21
**Anti-nuclear antibodies**	41 (100%)	28 (100%)	-

American College of Rheumatology (ACR) classification criteria reflect the clinical history of systemic lupus erythematosus (SLE) and are compared for patients who always displayed normal ability to degrade neutrophil extracellular traps (NETs) compared to patients who at least once displayed a decreased ability to degrade NETs. Asterisks indicate significant differences with the increased proportion highlighted by bold numbers.

**Figure 3:**
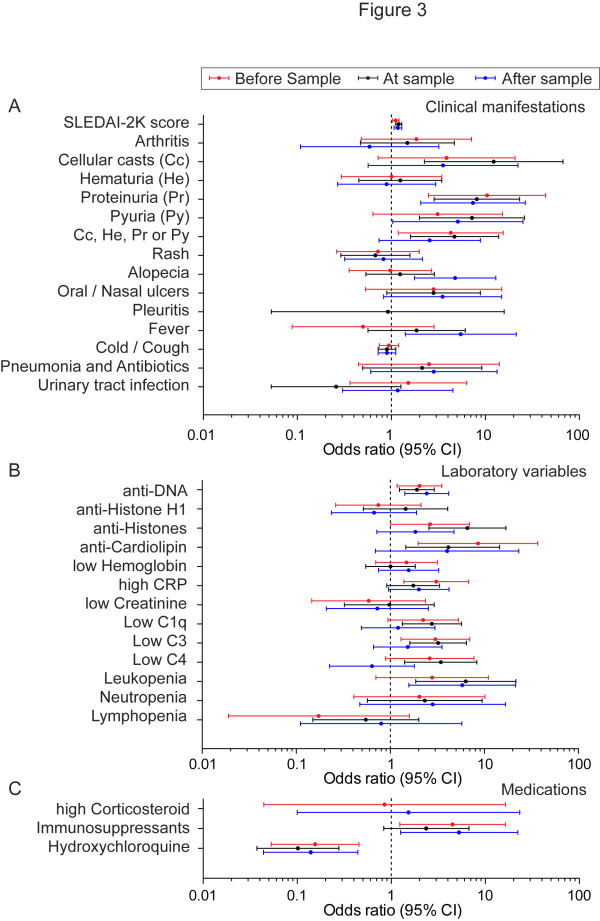
**Decreased ability to degrade NETs is associated with glomerulonephritis and altered laboratory variables**. **A-C**. Odds ratios for co-occurrence of indicated parameters in SLE patients with a decreased ability to degrade NETs (*n*: 26), divided into SLEDAI-2K and clinical manifestations (A), laboratory variables (B) and medications (C) two months before (red), at the same time (black) or two month after (blue) the time point with decreased ability to degrade NETs. The odds ratios are indicated with a dot connected to the 95% confidence intervals. All values that do not cross "1" are considered significant. NETs, neutrophil extracellular traps; SLE, systemic lupus erythematosus; SLEDAI-2K, Systemic Lupus Erythematosus Disease Activity Index 2000

We also investigated if any particular clinical manifestation, laboratory variable or treatment preceded, co-occurred or succeeded an episode with decreased NET degradation. To determine if a decreased degradation of NETs was associated with these variables that occurred either two months before sampling, at sampling, or two months after sampling, the odds ratios (OR) for the different parameters were calculated for sera with a decreased ability to degrade NETs. The parameters were divided into three categories; clinical manifestations derived from SLEDAI-2K, which includes mainly components of glomerulonephritis, symptoms from skin, joints and pleuritis as well as infections (Figure 3A), laboratory variables (Figure 3B) and medications (Figure 3C). Significant associations are also summarized in Table 3. A decreased ability to degrade NETs was associated with an increase of one point in SLEDAI-2K score with OR 1.1 (1.0 to 1.2) before sampling, 1.2 (1.1 to 1.3) at sampling and 1.2 (1.1 to 1.3) after sampling (Figure 3A). A decreased ability to degrade NETs was further associated with manifestations that are considered as a part of active glomerulonephritis consisting of cellular casts, hematuria, proteinuria, pyuria or all combined with OR 12.8 (2.3 to 67.1) for cellular casts at the time of sampling. The OR for the presence of proteinuria before, at the time of sampling, as well as after sampling, were 10.4 (2.49 to 43.9), 8.1 (2.9 to 23.1) and 7.4 (2.1 to 26.6), respectively. When pyuria was registered, OR was 7.2 (2.0 to 26.0) at the time of sampling and 5.1 (1.0 to 25.2) after sampling. Additional clinical manifestations that were not part of glomerulonephritis, but were associated with a decreased ability to degrade NETs, were both alopecia and fever with OR 4.8 (1.8 to 12.9) for alopecia and 5.5 (1.4 to 21.3) for fever to develop two months after an episode with decreased ability to degrade NETs.

**Table 3:** Parameters associated with decreased ability to degrade NETs

	approximately 60 days before	Simultaneously	approximately 60 days after
**Clinical**	Proteinuria	Proteinuria	Proteinuria
**manifestations**		Pyuria	Pyuria
		Cellular casts	Alopecia
			Fever
			
**Laboratory**	Anti-DNA	Anti-DNA	Anti-DNA
**variables**	Anti-Histones	Anti-Histones	Leukopenia
	Anti-Cardiolipin	Anti-Cardiolipin	
	Elevated CRP	Leukopenia	
	Low C3	Low C1q, C3 and C4	
			
**Medications**	No HC^a^	No HC^a^	No HC^a^
	Immunosuppressants		Immunosuppressants

Manifestations that occur significantly more frequently than expected, before, simultaneously or after the time point with decreased ability to degrade neutrophil extracellular traps (NETs).

^a^HC, hydroxychloroquine

Abnormal levels of some laboratory variables (Figure 3B) were associated with an increased OR preceding an episode with decreased ability to degrade NETs. These factors were elevated anti-DNA antibodies with OR 2.0 (1.2 to 3.5), anti-histone antibodies 2.6 (1.0 to 6.9), the presence of antibodies against cardiolipin 8.6 (2.0 to 37.1) and elevated CRP 3.1 (1.4 to 6.8). Furthermore, low levels of C3 and leukocytes (leukopenia) also preceded an episode with decreased degradation with OR 3.0 (1.3 to 7.0) and 5.8 (1.6 to 21.5), respectively. When analyzing laboratory variables at the same time-point as the episode of decreased degradation, the following ORs were obtained: anti-DNA antibodies 1.9 (1.3 to 2.9), anti-histone antibodies 6.6 (2.6 to 16.9) and anti-cardiolipin antibodies 4.1 (1.5 to 14.6). Reduced complement levels were also associated with an episode of decreased degradation with OR 2.8 (1.3 to 5.7) for low C1q, 3.2 (1.6 to 6.5) for low C3 and 3.4 (1.4 to 8.3) for low levels of C4. Leukopenia also generated an increased OR of 6.4 (1.9 to 21.6) at the time-point of decreased degradation of NETs.

Certain medications (Figure 3C) were also more commonly administered to patients whose sera had a decreased ability to degrade NETs. These patients were more often treated with immunosuppressants both before and after sampling with OR of 4.5 (1.2 to 16.4) and 5.3 (1.3 to 22.2), respectively. However, no association was seen at the time of decreased ability to degrade NETs. Patients on hydroxychloroquine treatment displayed an inverse correlation with decreased NET-degrading abilities with an OR of 0.2 (0.05 to 0.5) before, 0.1 (0.04 to 0.4) after and 0.1 (0.04 to 0.3) at the time of sampling.

## Discussion

In the current study we have extensively and in a truly prospective way, investigated the ability of SLE sera to degrade NETs with the goal of determining the overall importance of NET degradation for the disease. This was done in a longitudinal setup to reveal temporal associations as well as potential predictive values of the NET degrading ability. In the present study, 69 patients with SLE were followed for a median of 784 (mean 921) days with serum samples collected approximately every 60 days, generating a total of 1,074 measurements. We found that a decreased ability to degrade NETs was a rather common feature of SLE with 41% of the patients at least once, but commonly more than once, displaying a reduced ability to degrade NETs.

Among the patients that never displayed a reduced ability to degrade NETs, a clinical history of photosensitivity was more common whereas the patients who at least once had a reduced ability to degrade NETs more often displayed a clinical history of antibodies against DNA. Based on the cumulative classification criteria according to ACR, no other differences could be established between the two patient groups. ACR classification criteria reflect a cumulative clinical phenotype of SLE but not necessarily the picture at blood sampling. Therefore, SLEDAI-2K score was used to evaluate ongoing disease activity at the time of blood sampling. The cumulative nature of the ACR classification criteria may also explain why no difference was observed with a history of glomerulonephritis whereas active nephritis correlated strongly with a decreased ability to degrade NETs and has also been established in previous studies [7,8]. Nephritis in the ACR classification criteria may reflect events, which occurred long before the study was initiated and the NET-degrading ability at that time point is therefore not known. Most patients were included in the study several years after initial diagnosis.

During the study both the disease activity defined by SLEDAI-2K score as well as the ability to degrade NETs improved for most patients. This may be due to the fact that patients were normally recruited during active disease and an improvement would hence be expected due to efficient medication. We have previously shown that a decreased ability to degrade NETs is associated with a higher SLEDAI-2K score in patients, especially reflecting patients with nephritis [8], who also normally respond well to treatment. It is interesting to note that although no direct efforts were made to restore the ability to degrade NETs, such improvement was achieved. This indicates that a reduced ability to degrade NETs may be a secondary feature of SLE in a stage of active disease. Further, the results also indicate that regular follow-ups on patients with SLE may be beneficial in reducing SLE flares.

It has previously been shown that a decreased ability to degrade NETs is strongly associated with glomerulonephritis and the presence of antibodies directed against DNA and histones [7,8]. By studying the ability to degrade NETs in SLE in a longitudinal setting, particular clinical parameters could be associated with a reduced NET degrading ability (Table 3). We confirmed that patients with decreased degradation of NETs more often suffered from manifestations of glomerulonephritis. Some manifestations of glomerulonephritis were present both before and after the time-point with decreased NET-degradation, whereas cellular casts, as a strong indication of glomerular damage, only occurred during the episode with decreased degradation. Interestingly, alopecia and fever associated strongly only two months after an episode of decreased NET degradation. This indicates a predictive value and a potential diagnostic application but may also give insights into possible consequences of having a reduced ability to degrade NETs and its overall importance for pathogenesis.

Antibodies against epitopes present on NETs were further associated with a decreased ability to degrade NETs. NETs consist of chromatin, including DNA and histones, which are covered with antimicrobial enzymes [6]. Interestingly, elevated levels of antibodies against histone proteins were only associated with reduced ability to degrade NETs at the same time point as the decreased degradation occurred. Elevated and high levels (data not shown) of anti-DNA antibodies were, however, associated with decreased degradation before, at the same time, as well as after the episode of reduced degradation. This implies a more direct effect or response for antibodies against histones compared to the presence of antibodies against DNA that may have a slower turnover or response. Antibodies against DNA are heterogeneous and their detection varies according to the employed method and may explain the associations obtained. Notably, elevated antibody titers against cardiolipin also resulted in an elevated OR for reduced NET degradation both before and at sampling. The exact mechanisms for this remain to be elucidated since cardiolipin is not a known constituent of NETs but is, however, present in the cell membrane of dead cells [20].

Low complement levels are in some cases associated with disease flare [21] and are also associated with a decreased ability to degrade NETs. Interestingly, low C3 levels increased the OR for a reduced ability to degrade NETs within two months. Related to that observation, elevated levels of CRP also seem to precede an episode with a reduced ability to degrade NETs. Otherwise, low C1q and low C4 levels were only associated with decreased NET degrading ability at the time the decrease was observed. Patients on hydroxychloroquine treatment displayed lower ORs for an episode with reduced ability to degrade NETs. If the antimalarial therapeutics treatment itself has a direct effect of NET degradation is an interesting possibility; however, no effect on NET degradation was observed when hydroxychloroquine was added directly to serum compared to control (data not shown). The effect is, therefore, most likely secondary or possibly due to the fact that mainly patients with less severe form of SLE with mostly symptoms from skin and joints, excluding nephritis and presence of antibodies against DNA, were treated with hydroxychloroquine.

The frequency of sampling in the study only allows for detection of associations within two months (46 to 74 days) before or after a measurement and future studies with more frequent sampling could provide further details and resolution as in how fast the ability to degrade NETs changes over time and what parameters associate with those changes. On the other hand, for the majority of patients the current frequency proved narrow enough since it could take up to half a year to restore or improve a decreased ability to degrade NETs for many patients (Figure 2B).

Attempts to treat SLE with DNase-I have been made since the 1960s, unfortunately problems with antigenicity occurred and the trial discontinued [22]. However, new attempts were initiated with the expression of recombinant human DNase-I [23], which was also designed to withstand the presence of potential inhibitors [24]. No clear effects were observed in an early clinical trial in 1999 [23] although animal experiments had previously shown promising results [25]. Recombinant DNase-I has, however, been developed as an FDA approved drug and is used successfully in treating cystic fibrosis by inhalation [26]. This study reveals in which SLE patient group the treatment to restore NET degrading ability may be most beneficial.

## Conclusion

The present study confirms previously published results of manifestations that are directly associated with a decreased ability to degrade NETs. Further, it expands, for the first time, on previous findings in a longitudinal setting showing that a decreased ability to degrade NETs is a common (41% of included patients) feature of SLE. Decreased ability to degrade NETs is also associated with a history of anti-DNA antibodies as well as active glomerulonephritis and high titers of antibodies directed against histone proteins and DNA at the time of decreased degradation. Additional associations indicate that a reduced ability to degrade NETs may precede fever and alopecia. Altogether our findings support the importance of maintaining the ability to degrade NETs in SLE and may benefit patients as a diagnostic application.

## Abbreviations

ACR: American college of rheumatology; CRP: C-reactive protein; NETs: neutrophil extracellular traps; OR: odds ratio; PBS: phosphate-buffered saline; SLE: systemic lupus erythematosus; SLEDAI-2K: SLE disease activity index 2000.

## Competing interests

The authors declare that they have no competing interests.

## Authors' contributions

JL, MM and BG performed experiments. JL wrote the manuscript together with AMB and AAB, who both also supervised the study. AJ maintained and collected patient data. JAN analyzed the data statistically. All authors read and edited the manuscript.
